# Structural proteins of West Nile virus are a major determinant of infectious particle production and fitness in astrocytes

**DOI:** 10.1099/vir.0.065474-0

**Published:** 2014-09

**Authors:** Katherine L. Hussmann, Rianna Vandergaast, Kang Zheng, Lisa I. Hoover, Brenda L. Fredericksen

**Affiliations:** 1 Department of Cell Biology and Molecular Genetics, University of Maryland, College Park, MD 20742, USA; 2 Maryland Pathogen Research Institute, University of Maryland, College Park, MD 20742, USA

## Abstract

The molecular basis for the increased resistance of astrocytes to a non-neuropathogenic strain of West Nile virus (WNV), WNV-MAD78, compared with the neuropathogenic strain WNV-NY remains unclear. Here, we demonstrated that the reduced susceptibility of astrocytes to WNV-MAD78 is due to a combination of both cellular activities as well as viral determinants. Analyses of the viral particle indicated that astrocyte-derived WNV-MAD78 particles were less infectious than those of WNV-NY. Additionally, inhibition of cellular furin-like proteases increased WNV-MAD78 infectious particle production in astrocytes, suggesting that high levels of furin-like protease activity within these cells acted in a cell- and strain-specific manner to inhibit WNV-MAD78 replication. Moreover, analysis of recombinant viruses indicated that the structural proteins of WNV-MAD78 were responsible for decreased particle infectivity and the corresponding reduction in infectious particle production compared with WNV-NY. Thus, the composition of the WNV virion was also a major determinant for viral fitness within astrocytes and may contribute to WNV propagation within the central nervous system. Whether the WNV-MAD78 structural genes reduce virus replication and particle infectivity through the same mechanism as the cellular furin-like protease activity or whether these two determinants function through distinct pathways remains to be determined.

## Introduction

West Nile virus (WNV) is a neurotropic member of the genus *Flavivirus* that has emerged recently as a significant threat to human health. Historically, most WNV infections have been asymptomatic or associated with a mild febrile illness known as West Nile fever. However, recent outbreaks in the Western hemisphere have been characterized by a marked increase in the percentage of neurological infections. Since 1999, infections have resulted in >17 000 cases of severe neurological disease in the USA alone (http://www.cdc.gov/westnile/statsMaps/), making WNV the leading cause of arboviral encephalitis in North America. The viral factors responsible for the increased incidence of neurological disease associated with these recent outbreaks remain poorly understood.

The WNV life cycle begins with attachment of the virus to the cell surface. Whilst attachment in some cell types is mediated by interactions between the viral envelope (E) protein and integrin α_V_β_3_ (Davis *et al.*, 2006a), binding of *N*-linked glycans present on the viral glycoproteins to the lectin-binding proteins DC-SIGN and DC-SIGNR contributes to attachment in other cells (Davis *et al.*, 2006b; Martina *et al.*, 2008). Following attachment, virus particles are internalized by receptor-mediated endocytosis and transported to endosomes (Brinton, 2002). Exposure to the low pH of the endosome triggers fusion of the viral envelope with the endosomal membrane, thereby releasing the viral genome into the cytoplasm. The incoming viral genome serves as a template for both translation of viral proteins and replication. Once sufficient levels of viral proteins and nascent RNA genomes accumulate, immature particles, which consist of the viral genome surrounded by the capsid (C), precursor membrane (prM) and E proteins, assemble at the endoplasmic reticulum membrane. During egress through the secretory pathway, cleavage of prM by furin-like proteases promotes conformational changes to the global structure of the virion, which leads to the formation of the mature virus particle. As the virion is released into the extracellular milieu, the cleaved precursor portion dissociates, completing the maturation process.

In many cell types, cleavage of prM is inefficient, resulting in the production of particles with maturation states ranging from fully immature to fully mature (Nelson *et al.*, 2008; Pierson & Diamond, 2012). Early studies suggested that complete maturation is necessary to render flavivirus particles infectious (Stadler *et al.*, 1997). However, recent studies have demonstrated that not only are virions retaining uncleaved prM infectious (Colpitts *et al.*, 2011; Mukherjee *et al.*, 2011; Nelson *et al.*, 2008; Zybert *et al.*, 2008), but that complete maturation may be detrimental to WNV replication under certain circumstances (Zybert *et al.*, 2008). Whilst most strains of WNV contain a glycosylation site in both E and the precursor portion of prM, some non-pathogenic strains lack the E glycosylation site. For these non-pathogenic strains, maturation removes all *N*-linked glycans from the virion, abrogating attachment to the cell via C-type lectins and reducing infectivity on cells expressing DC-SIGN or DC-SIGNR (Davis *et al.*, 2006b). Thus, virus maturation status and the presence of *N*-linked glycans on the virion may influence WNV pathogenesis. Indeed, several studies have demonstrated that the glycosylation state of the viral particle contributes to the pathogenicity of WNV (Beasley *et al.*, 2005; Botha *et al.*, 2008; Hanna *et al.*, 2005; Hobson-Peters *et al.*, 2008; Scherret *et al.*, 2001).

WNV primarily enters the central nervous system (CNS) by crossing the blood–brain barrier (BBB) (Cho & Diamond, 2012; Hussmann *et al.*, 2013; Roe *et al.*, 2012; Verma *et al.*, 2009). The BBB is a highly restrictive barrier that protects the CNS from aberrant immune responses and pathogens in the periphery (Abbott, 2002). Interactions between WNV and components of the BBB therefore have the potential to impact neuropathogenesis significantly. The BBB comprises endothelial cells, which line the cerebral vasculature, and astrocytes, which are in direct contact with the endothelial cells. Astrocytes play a central role in maintaining homeostasis within the CNS by regulating the integrity of the BBB as well as the uptake of excess neurotransmitters and other extracellular factors that may perturb neurotransmission (Abbott, 2002). Moreover, evidence suggests that WNV replication in astrocytes may influence the physiological outcome of viral infections of the CNS (Roe *et al.*, 2012; Verma *et al.*, 2010; Wang *et al.*, 2004).

In astrocytes, replication of an avirulent lineage 2 African isolate, WNV-MAD78, is delayed and reduced compared with that of a highly virulent lineage 1 isolate, WNV-NY (Hussmann *et al.*, 2013). Here, we examined the viral and cellular factors responsible for this differential replication. We observed that within astrocytes, WNV-MAD78 infectious particle production was reduced by the high activity of furin-like proteases. As maturation enhances WNV infectious particle production in other cell lines (Stadler *et al.*, 1997), this finding suggests that furin-dependent restriction of WNV-MAD78 replication is an astrocyte-specific mechanism. Astrocyte-derived WNV-MAD78 particles were also significantly less infectious than those of WNV-NY. Characterization of a series of WNV-NY and WNV-MAD78 chimeric viruses suggested that the structural proteins were the primary viral components that dictated particle infectivity and infectious particle production. Thus, WNV structural proteins were principal determinants of viral propagation within astrocytes.

## Results

### WNV-MAD78 replication in astrocytes is not restricted by a secreted host factor

**Fig. 1.**
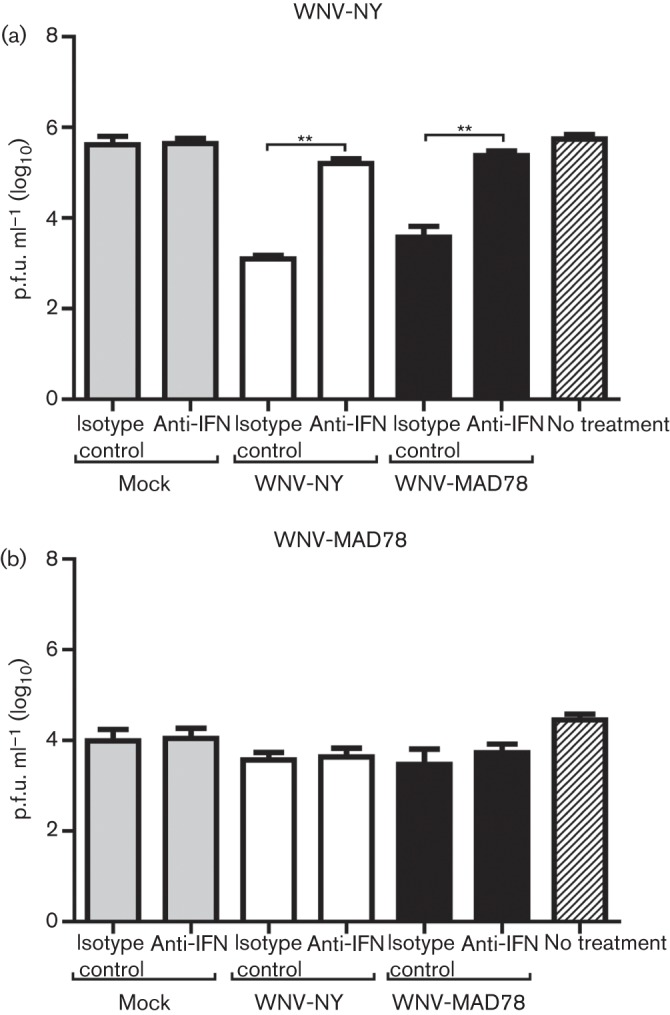
Examination of the role of a secreted factor in limiting WNV infection in astrocytes. HBCAs were pretreated (6 h) with UV-inactivated supernatants recovered from mock- or WNV-infected HBCAs (with or without antibodies to type I IFN or corresponding isotype control) and infected with (a) WNV-NY or (b) WNV-MAD78 (m.o.i. 0.01). After 1 h at 37 °C, the viral inoculum was replaced with UV-inactivated supernatants (with or without antibodies to type I IFN or corresponding isotype control). Culture supernatants were collected at 24 h post-infection and viral titres determined by plaque assays on Vero cells. Values represent the mean±se p.f.u. ml^–1^ in supernatant from three independent experiments. **P<0.01.

In astrocytes, the reduction in WNV-MAD78 replication compared with that of WNV-NY is due in part to IFN-independent programmes (Hussmann *et al.*, 2013). To determine if another secreted factor contributes to the reduction in WNV-MAD78 replication, we assessed the inhibitory potential of supernatant collected from WNV-infected astrocytes. Human brain cortical astrocytes (HBCAs) were treated with UV-inactivated supernatants recovered from mock-, WNV-NY- or WNV-MAD78-infected HBCAs. Whilst treatment of HBCAs with supernatants from mock-infected cells had no effect on viral titres, supernatants recovered from WNV-infected cells significantly reduced WNV-NY replication (Fig. 1a). However, neutralizing antibodies to IFN-α and IFN-β completely abrogated the inhibitory capacity of the WNV supernatants, indicating that the effect was due to type I IFN and not another secreted factor. Unlike WNV-NY, WNV-MAD78 infectious particle production was not affected by treatment with supernatants from WNV-infected cultures in either the presence or absence of antibodies to type I IFN (Fig. 1b). These results were consistent with the finding that in astrocytes, IFN plays a role in restricting the replication of WNV-NY but not WNV-MAD78 (Hussmann *et al.*, 2013). Furthermore, our findings indicated that the lower level of WNV-MAD78 replication compared with that of WNV-NY in HBCAs was not due to the antiviral effects of a secreted factor.

### Astrocyte-derived WNV-MAD78 particles are less infectious than those of WNV-NY

**Fig. 2.**
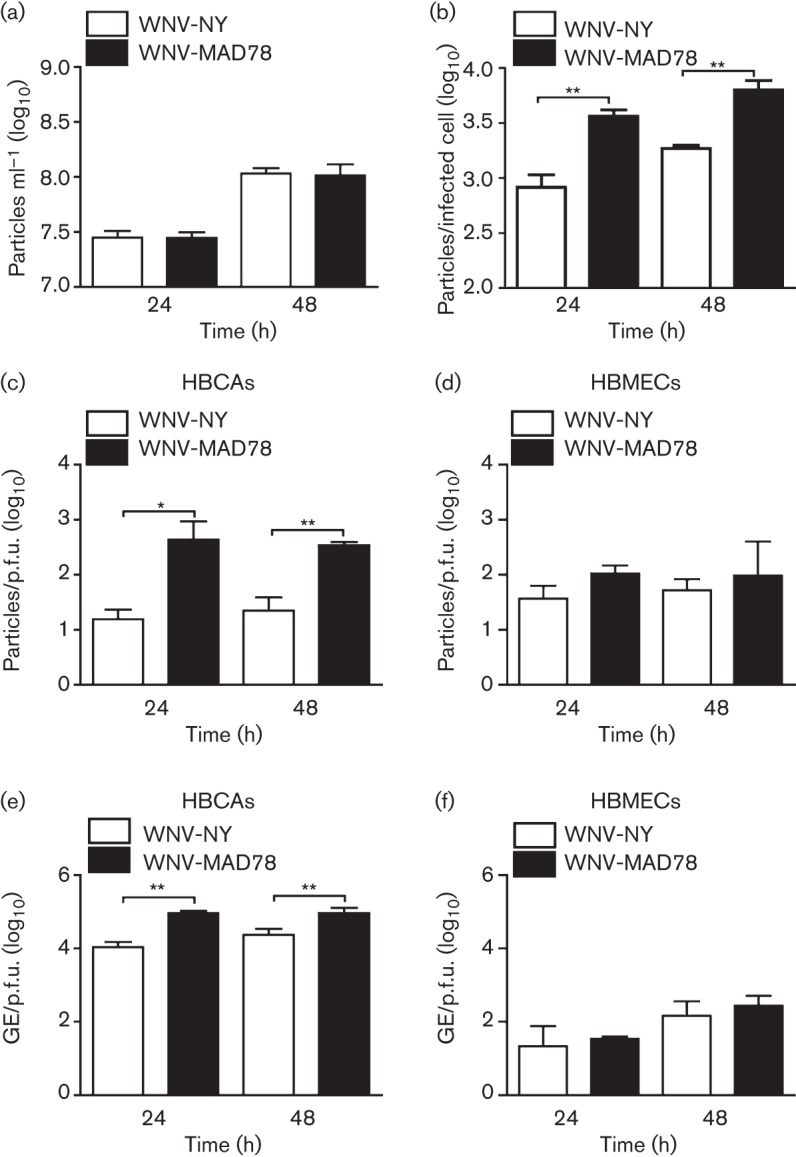
Infectivity of HBCA-derived particles. HBCAs were infected with WNV-NY or WNV-MAD78 (m.o.i. 0.01). (a) Total viral particle production at 24 and 48 h post-infection. Culture supernatants were removed at the indicated times and particle levels quantified using the ViroCyt virus counter. Values represent the mean±se number of particles ml^–1^ of three independent experiments. (b) Particle production per infected cell was determined by dividing the total number of viral particles present in the culture supernatant as determined in (a) by the total number of infected cells as determined by flow cytometry. Values represent mean±se of three independent experiments. **P<0.01. (c–f) Infectivity of astrocyte-derived (c, e) and HBMEC-derived (d, f) WNV particles. (c, d) The concentration of total virus particles and infectious particles was determined using the ViroCyt virus counter and plaque assays on Vero cells, respectively. Values represent the mean±se ratio of total particles compared with infectious particles from three independent experiments. *P<0.05, **P<0.01. (e, f) The concentration of viral genomes (GE) and infectious particles was determined using qRT-PCR and plaque assays on Vero cells, respectively. Values represent the mean±se ratio of viral genomes compared with infectious particles from three independent experiments. **P<0.01.

As WNV-MAD78’s decreased replication compared with that of WNV-NY was not due to a secreted host factor, we hypothesized that it may be a result of impaired WNV-MAD78 particle production or infectivity. Therefore, we compared WNV-NY and WNV-MAD78 particle production using a virus counter, which utilized two dyes in a flow-cytometry-based system to detect nucleic acid and protein simultaneously, thereby excluding empty particles and cellular debris from analysis. Using this approach, we found that similar levels of total viral particles were present in supernatants from WNV-NY- and WNV-MAD78-infected astrocytes at 24 and 48 h post-infection (Fig. 2a). Furthermore, when the total number of cells infected with each virus was determined by flow cytometry, we found that WNV-MAD78-infected HBCAs produced more particles per infected cell than HBCAs infected with WNV-NY (Fig. 2b). Comparison of the physical counts of total virus particles obtained from the virus counter and the biological counts of infectious particles determined by plaque assay on Vero cells indicated that the particle/p.f.u. ratio was higher for WNV-MAD78 than WNV-NY (Fig. 2c). Thus, WNV-MAD78 particles produced by HBCAs were less infectious than WNV-NY particles produced by HBCAs. To determine if the differential infectivity was specific to astrocyte-derived virus particles, we examined viral particles produced by human brain microvascular endothelial cells (HBMECs) – a cell line in which WNV-MAD78 and WNV-NY replicate at similar rates and to similar levels (Hussmann *et al.*, 2013). The particle/p.f.u. ratios for WNV-NY and WNV-MAD78 were similar in HBMECs (Fig. 2d). To confirm these results, we also determined the level of genome equivalents per p.f.u. in the supernatants recovered from WNV-infected HBCAs and HBMECs (Fig. 2e, f). Consistent with our findings using the virus counter, HBCA-derived WNV-MAD78 exhibited lower infectivity compared with HBCA-derived WNV-NY, whilst particles produced by HBMECs had similar levels of infectivity. Together, our findings suggested that the reduction in WNV-MAD78 replication in HBCAs was due in part to a decrease in particle infectivity.

### Differential infectivity of WNV-NY and WNV-MAD78 is not due to variation in particle stability

**Fig. 3.**
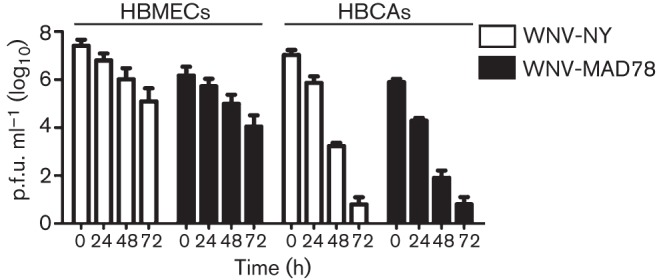
Stability of HBCA- and HBMEC-derived WNV particles. Culture supernatants recovered from WNV-NY- or WNV-MAD78-infected HBMECs (m.o.i. 0.1) and HBCAs (m.o.i. 0.01) at 48 h post-infection were incubated at 37 °C. Aliquots were removed at the indicated times and infectious particles quantified by plaque assays on Vero cells. Values represent the mean±se p.f.u. ml^–1^ of at least two independent experiments.

It is possible that WNV-MAD78’s reduced infectivity compared with WNV-NY in HBCAs was due to variations in particle stability. To test this, we assessed HBMEC- and HBCA-derived WNV-MAD78 and WNV-NY particle stability at 37 °C. The infectivity of WNV-NY and WNV-MAD78 particles produced by HBMECs decreased ~100-fold over 72 h (Fig. 3). In contrast, the infectivity of both WNV-NY and WNV-MAD78 particles produced by HBCAs was reduced nearly 10^6^-fold under the same conditions. Thus, WNV particles produced by HBCAs were significantly less stable than WNV particles produced by HBMECs. Nevertheless, the rate at which WNV-MAD78 particles lost infectivity over time was not significantly different from that of WNV-NY particles in either cell line (Fig. 3), suggesting that the differential replication of these viruses in HBCAs was not due to the enhanced stability of WNV-NY particles compared with that of WNV-MAD78 particles.

### Furin limits WNV-MAD78 infectious particle production in astrocytes

**Fig. 4.**
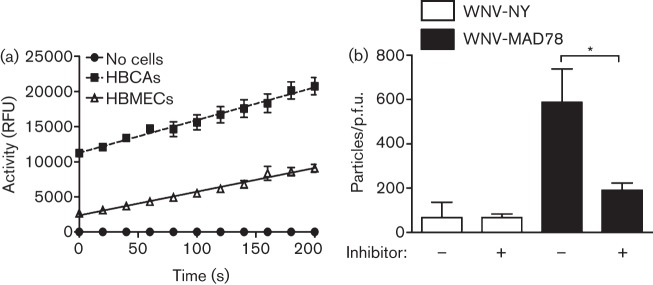
Effect of furin-like protease activity on infectious particle production in astrocytes. (a) Furin activity in confluent monolayers of HBCAs and HBMECs. HBCA and HBMEC monolayers were lysed in assay buffer. The furin substrate pERTKR-amino-methylcoumarin (100 µM) was added to equal cellular amounts of lysate and fluorescence was measured every 20 s for 10 min. Values represent the mean±se relative fluorescence units (RFU) of at least three independent experiments. (b) HBCAs were infected with WNV-NY or WNV-MAD78 (m.o.i. 0.01) in the presence or absence of the furin inhibitor Dec-RVKR-CMK (50 µM). Culture supernatants were collected at 48 h post-infection. The concentration of total virus particles and infectious particles was determined using the ViroCyt virus counter and plaque assays on Vero cells, respectively. Values represent the mean±se number of particles/p.f.u. ml^–1^ of at least three independent experiments. *P<0.05.

A key factor influencing flavivirus particle infectivity is the maturation state of the exocytosed particles (Pierson & Diamond, 2012; Stadler *et al.*, 1997). For WNV, maturation efficiency is mediated by cellular furin-like serine proteases. We hypothesized that the degree of furin activity within a cell may impact WNV-MAD78 infectious particle production more than WNV-NY infectious particle production. Thus, we predicted that furin activity in cell types exhibiting similar levels of WNV-NY and WNV-MAD78 infectious particle production would be higher than in cells where WNV-MAD78 infectious particle production was impaired. However, when we compared HBCAs and HBMECs, the level of furin activity was approximately fourfold higher in HBCAs than in HBMECs (Fig. 4a). Therefore, furin activity correlated inversely with WNV-MAD78 infectious particle production. To assess directly if furin activity in astrocytes affected WNV-MAD78 replication, we compared WNV-NY and WNV-MAD78 particle to p.f.u. ratio in the presence and absence of furin inhibitors. Addition of furin inhibitors had no effect on WNV-NY particle to p.f.u. ratio in HBCAs (Fig. 4b). In contrast, WNV-MAD78 particle to p.f.u. ratio increased in the presence of the furin inhibitors, suggesting that the high level of furin-like protease activity in astrocytes contributed to the decrease in WNV-MAD78 infectious particle production compared with that of WNV-NY.

### Glycosylation status of prM and E does not affect WNV replication in astrocytes

**Fig. 5.**
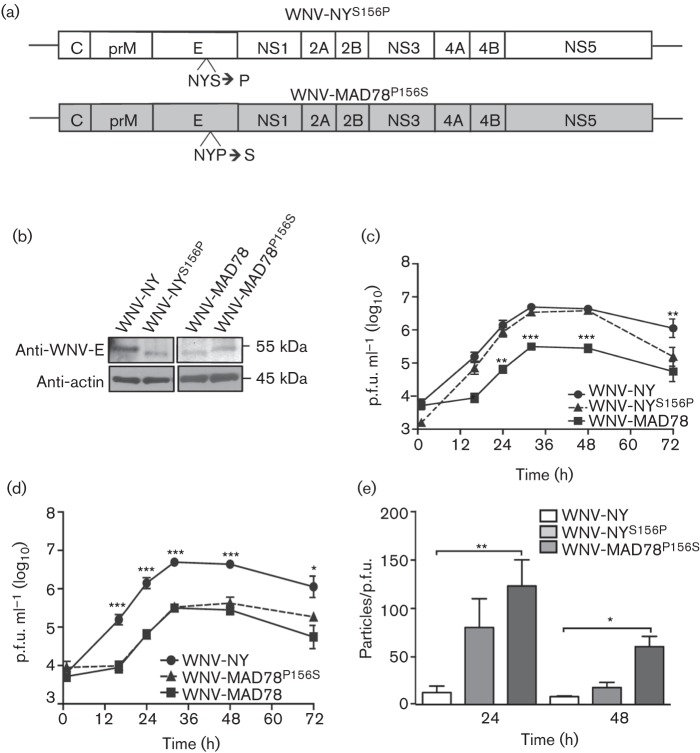
Replication of WNV-NY^S156P^ and WNV-MAD78^P156S^ in HBCAs. (a) Schematic representation of WNV-NY^S156P^ and WNV-MAD78^P156S^. (b) Immunoblot analysis of WNV-NY^S156P^ and WNV-MAD78^P156S^ E proteins. HBCAs were infected (m.o.i. 0.01) with WNV-NY, WNV-MAD78, WNV-NY^S156P^ or WNV-MAD78^P156S^. Cell lysates collected 48 h post-infection were subjected to SDS-PAGE (8–16 % denaturing gel) and immunoblot analysis using anti-WNV-E (top) and anti-actin (bottom). (c, d) Replication of WNV-NY^S156P^ and WNV-MAD78^P156S^ in HBCAs. HBCAs were infected (m.o.i. 0.01) with WNV-NY, WNV-MAD78 and WNV-NY^S156P^ (c) or WNV-MAD78^P156S^ (d). At the indicated times, culture supernatants were collected and viral titres determined by plaque assay on Vero cells. Values represent the mean±se p.f.u. ml^–1^ from three independent experiments. Statistical significance comparing replication of both chimeras in relation to WNV-NY (indicated above the line representing WNV-NY) and WNV-MAD78 (indicated above the line representing WNV-MAD78) is shown. *P<0.05, **P<0.01, ***P<0.005. (e) Infectivity of astrocyte-derived WNV-NY^S156P^ and WNV-MAD78^P156S^ particles. The concentration of total virus particles and infectious particles (c, d) was determined using the ViroCyt virus counter and plaque assays on Vero cells, respectively. Values represent the mean±se number of particles/p.f.u. ml^–1^ of at least three independent experiments. Infectivity of both recombinant viruses was compared with the infectivity of WNV-NY as determined in Fig. 2(c). *P<0.05, **P<0.01.

As WNV-MAD78 is only glycosylated on the precursor portion of prM, cleavage of prM by furin-like proteases reduces, if not eliminates, the level of *N*-linked glycans on WNV-MAD78 particles. Thus, driving viral particle maturation to completion may impair WNV-MAD78’s ability to bind and infect astrocytes, which express DC-SIGN and DC-SIGNR (data not shown and Davis *et al.*, 2006b; Martina *et al.*, 2008). WNV-NY, in contrast, may retain its ability to bind DC-SIGN and DC-SIGNR after maturation due to the second glycosylation site within E. Variations in glycosylation may therefore account for the differential effects of furin-like protease activity on WNV-NY and WNV-MAD78 in HBCAs. To assess the effect of glycosylation of the E protein on WNV replication in astrocytes, we generated recombinant viruses in which the glycosylation sites within the E proteins of WNV-MAD78 and WNV-NY were exchanged (Fig. 5a). The glycosylation status of the recombinant viruses was confirmed by immunoblot analysis (Fig. 5b). Neither introduction of a glycosylation site into the E protein of WNV-MAD78 (WNV-MAD78^P156S^) nor removal of the glycosylation site from WNV-NY (WNV-NY^S156P^) altered viral replication rates or peak viral levels (Fig. 5c, d). The infectivity of WNV-MAD78^P156S^ was similar to parental to WNV-MAD78 and was reduced compared with WNV-NY at 24 and 48 h post-infection (Fig. 5e), indicating that introduction of the glycosylation site did not increase the infectivity of WNV-MAD78 viral particles. Furthermore, removal of the glycosylation site from WNV-NY had no effect on particle infectivity at 48 h post-infection. Whilst the infectivity of WNV-NY^S156P^ tended to be lower than parental WNV-NY at 24 h post-infection, this difference did not reach statistical significance. Therefore, *N*-linked glycans on the WNV envelope were not a major determinant of WNV fitness in astrocytes.

### WNV structural proteins contribute to infectious particle production in astrocytes

**Fig. 6.**
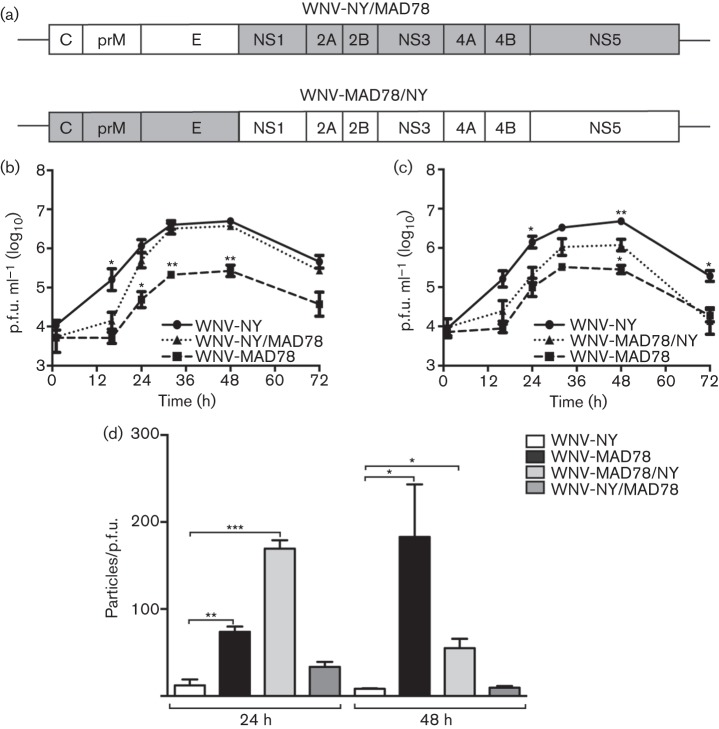
Replication of WNV-NY/MAD78 and WNV-MAD78/NY in HBCAs. (a) Schematic representation of WNV-NY/MAD78 and WNV-MAD78/NY. Regions derived from WNV-NY are in white and regions from WNV-MAD78 are in grey. (b–d) HBCAs were infected with WNV (m.o.i. 0.01). (b, c) Growth curves of WNV-NY, WNV-MAD78 and WNV-NY/MAD78 (b) or WNV-MAD78/NY (c). Culture supernatants were collected at the indicated times and viral titres determined by plaque assay on Vero cells. Values represent the mean±se p.f.u. ml^–1^ of at least three independent experiments. Statistical significance comparing replication of both chimeras in relation to WNV-NY (indicated above the line representing WNV-NY) and WNV-MAD78 (indicated above the line representing WNV-MAD78) is shown. *P<0.05, **P<0.01. (d). Infectivity of astrocyte-derived WNV-NY, WNV-MAD78, WNV-MAD78/NY and WNV-NY/MAD78 particles. The concentration of total virus particles and infectious particles (b, c) was determined using the ViroCyt virus counter and plaque assays on Vero cells, respectively. Values represent the mean±se ratio of total particles to infectious particles from three independent experiments. *P<0.05, **P<0.01, ***P<0.005.

To identify viral components responsible for the differential replication and infectivity of WNV-MAD78 and WNV-NY, we generated chimeric viruses of these two strains (Fig. 6a). Replication of the chimera containing the WNV-NY structural and WNV-MAD78 non-structural genes (WNV-NY/MAD78) was delayed compared with that of WT WNV-NY (Fig. 6b), although overall peak levels of infectious particles were similar. The particle/p.f.u. ratios of WNV-NY/MAD78 and WNV-NY were also similar, indicating that the non-structural genes did not influence the infectivity of the resulting virus particles (Fig. 6d). Consistent with this finding, the chimera composed of the WNV-MAD78 structural and WNV-NY non-structural genes (WNV-MAD78/NY) replicated with similar kinetics to WT WNV-MAD78. However, slightly higher levels of WNV-MAD78/NY infectious particles were detected at 48 h post-infection (Fig. 6c), which corresponded with a modest increase in infectivity of the virus particles produced at this time point (Fig. 6d).

**Fig. 7.**
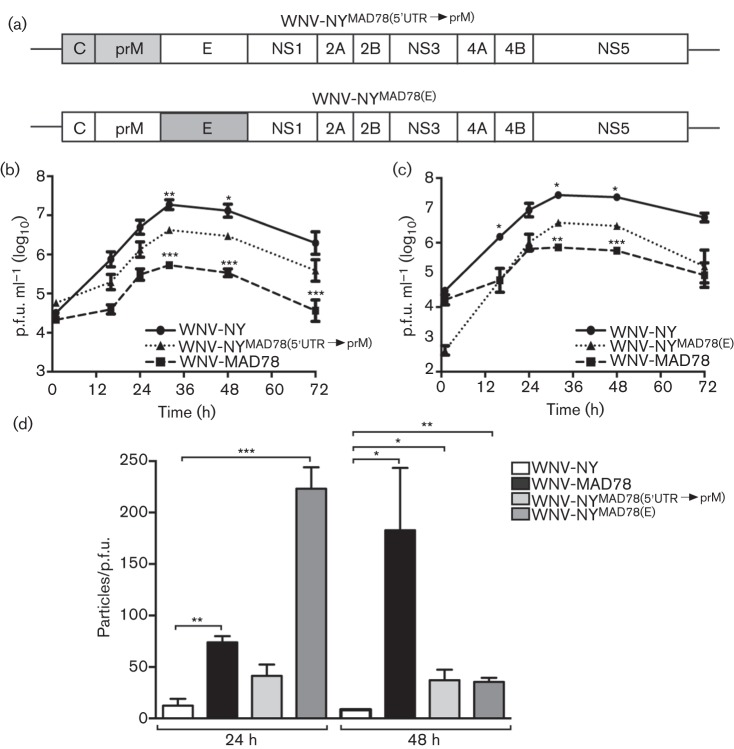
Replication of WNV-NY^MAD78(5′UTR→prM)^ and WNV-NY^MAD78(E)^ in HBCAs. (a) Schematic representation of WNV-NY^MAD78(5′UTR→prM)^ and WNV-NY^MAD78(E)^. Regions derived from WNV-NY are in white and regions from WNV-MAD78 are in grey. (b–d) HBCAs were infected with the indicated viruses (m.o.i. 0.01). (b, c) Growth curves of WNV-NY, WNV-MAD78 and WNV-WNV-NY^MAD78(5′UTR→prM)^ (b) or WNV-NY^MAD78(E)^ (c). Culture supernatants were collected at the indicated times and viral titres determined by plaque assay on Vero cells. Values represent the mean±se p.f.u. ml^–1^ of at least three independent experiments. Statistical significance comparing replication of both chimeras in relation to WNV-NY (indicated above the line representing WNV-NY) and WNV-MAD78 (indicated above the line representing WNV-MAD78) is shown. *P<0.05, **P<0.01, ***P<0.005. (d) Infectivity of astrocyte-derived WNV-NY, WNV-MAD78, WNV-NY^MAD78(5′UTR→prM)^ and WNV-NY^MAD78(E)^ particles. The concentration of total virus particles and infectious particles (b, c) was determined using the ViroCyt virus counter and plaque assays on Vero cells, respectively. Values represent the mean±se ratio of total particles to infectious particles from three independent experiments. *P<0.05, **P<0.01 ***P<0.005.

Our findings indicated that the deficiency in WNV-MAD78 multiplication in astrocytes mapped primarily to the structural genes. To further delineate the viral determinants involved, we generated chimeras in which the E gene or 5′UTR, C and prM genes of WNV-NY were replaced with the respective genes from WNV-MAD78 [designated WNV-NY^MAD78(E)^ and WNV-NY^MAD78(5′UTR→prM)^] (Fig. 7a). Peak viral titres of WNV-NY^MAD78(5′UTR→prM)^ and WNV-NY^MAD78(E)^ were significantly lower than those of WT WNV-NY (Fig. 7b, c). Although the 5′UTR of WNV-MAD78 has a single nucleotide deletion compared with that of WNV-NY, the 5′UTR does not affect WNV replication in astrocytes (Hussmann *et al.*, 2014). Therefore, the WNV-MAD78 C and/or prM proteins were responsible for WNV-NY^MAD78(5′UTR→prM)^’s differential replication. The decreased level of infectious particle production of WNV-NY^MAD78(5′UTR→prM)^ and WNV-NY^MAD78(E)^ likewise corresponded with lower infectivity of the viral particles, as observed previously for the recombinant virus containing all the WNV-MAD78 structural genes (Fig. 6c, d). WNV-NY^MAD78(E)^ exhibited a significantly higher particle/p.f.u. ratio than either WT WNV-NY or WNV-NY^MAD78(5′UTR→prM)^ at 24 h post-infection, suggesting that the E protein has a greater impact than C or prM on the infectivity of WNV particles at early times during infection (Fig. 7d). Nevertheless, the cognate structural proteins of WNV-NY were necessary for optimal infectious particle production in astrocytes.

## Discussion

The results of this study suggest that the impairment of WNV-MAD78 replication in astrocytes is due in part to the increased production of defective particles compared with WNV-NY. Moreover, both viral and cellular factors contribute to this lower infectivity. Characterization of chimeras of WNV-MAD78 and WNV-NY indicated that the infectivity of astrocyte-derived WNV particles corresponds to the cognate structural proteins. Examination of the cellular factors involved in the astrocyte-specific restriction to WNV-MAD78 replication indicated that high levels of furin-like protease inhibited WNV-MAD78, but not WNV-NY, infectious particle production. In contrast, the classical innate antiviral pathways did not contribute to the reduced level of WNV-MAD78 replication.

Astrocytes are the most abundant cell type within the CNS and play a key role in regulating CNS homeostasis (Abbott, 2002). Infected astrocytes have been detected in fatal human cases of WNV encephalitis, suggesting that these cells are also targeted by WNV *in vivo* (van Marle *et al.*, 2007). Various secreted factors, such as the chemokine ligand CCL5, IFN, nitrous oxide, reactive nitrogen species and exosomes, play a role in restricting infection of astrocytes by several neurotropic viruses including herpes virus, human immunodeficiency virus and Junin virus (Gómez *et al.*, 2003; Hu *et al.*, 2012; Li *et al.*, 2011). Although WNV-infected astrocytes express IFN-β, CCL5 and additional chemokines (Hussmann & Fredericksen, 2014; Hussmann *et al.*, 2013; Kumar *et al.*, 2010), our analyses indicated that secreted factors are not involved in inhibiting WNV-MAD78 infectious particle production (Fig. 1) (Hussmann *et al.*, 2013). However, IFN-β is responsible for limiting WNV-NY replication in astrocytes (Hussmann *et al.*, 2013). Thus, although WNV infection triggers many of the same antiviral programmes as other neurotropic viruses, the effect of these responses in controlling infection is both virus and strain specific.

As obligate intracellular parasites, viruses utilize various cellular components throughout their life cycle. As a result, differential interactions with basic cellular functions may account for strain-to-strain variations in infectivity. One cellular factor that modulates WNV infectivity is furin-like protease activity, which mediates maturation of the virus particle. In cell types representative of a peripheral infection, increased levels of furin-like protease activity enhance WNV particle maturation and infectivity (Davis *et al.*, 2006b; Mukherjee *et al.*, 2011; Pierson & Diamond, 2012). However, we found that furin activity inversely correlated with the susceptibility of cell types comprising the neurovascular unit to WNV infection, as more permissive HBMECs (Hussmann *et al.*, 2013) contained lower levels of furin activity than the more restrictive astrocytes. Moreover, inhibition of furin enhanced infectious particle production of WNV-MAD78 in astrocytes, demonstrating that furin is one of the factors responsible for the increased resistance of this cell type to WNV-MAD78. For many viruses, maturation of viral structural proteins is mediated by furin-like proteases. As such, inhibitors of furin have been proposed as a novel class of broad-spectrum antiviral compounds (Becker *et al.*, 2012). However, our findings suggest that in the case of WNV, furin’s role in mediating particle infectivity may be more complex. The effectiveness of furin inhibitors as antiviral compounds against WNV is therefore likely to be cell type dependent and may in fact augment WNV infections in the CNS.

One result of furin processing of WNV particles is a decrease in the level of *N*-linked glycans on the virion surface. As glycosylated proteins on viral particles can facilitate binding to the host cell through attachment factors such as DC-SIGN and DC-SIGNR (Davis *et al.*, 2006b; Martina *et al.*, 2008), a decrease in glycosylated proteins may reduce infectivity. We found that the presence or absence of an *N*-linked glycosylation site in the E protein of WNV-MAD78 or WNV-NY had no effect on the growth of either strain in astrocytes. Therefore, the inhibitory effect of furin is independent of glycosylation status. Moreover, this finding suggests that attachment of WNV to astrocytes is largely carbohydrate independent. Indeed, although astrocytes express DC-SIGN and DC-SIGNR (Deiva *et al.*, 2006 and data not shown), blocking antibodies to these receptors had no effect on infectious particle production (data not shown). Several studies have shown that the glycosylation state of the viral particle contributes to the pathogenicity of WNV (Beasley *et al.*, 2004, 2005; Hanna *et al.*, 2005; Hobson-Peters *et al.*, 2008; Scherret *et al.*, 2001). However, the basis for this difference in pathogenicity remains unclear. As both WNV-MAD78 and WNV-NY replicate in other cells of the CNS (i.e. neurons and endothelial cells) at equivalent rates (Hussmann *et al.*, 2013) and glycosylation did not affect WNV replication within astrocytes (Fig. 5), the glycosylation site on E may not play a role in regulating infection of the primary cell types within the CNS. Instead, within the CNS, the pathogenic effect of the glycosylation state may be due to alteration of infection and/or stimulation of immune cells.

Analysis of chimeric viruses of WNV-NY and WNV-MAD78 indicated that infectious particle production and particle infectivity corresponded primarily to the structural, not non-structural, components of the viruses. However, the slightly higher levels of WNV-MAD78/NY compared with WT WNV-MAD78 at 48 h post-infection suggest that the non-structural proteins may influence infectious particle production later during infection. In addition, viral fitness was not dependent on a single structural component, as further analyses indicated that WNV-NY chimeras containing either C and prM or the E protein of WNV-MAD78 exhibited decreased infectious particle production and infectivity. Whilst the precise defect in astrocyte-derived WNV-MAD78 particles is not known, the involvement of the viral structural proteins suggests that these virions are inefficient at one or more steps in the entry process. The structural proteins define the composition of the virion, and therefore are major determinants for permissiveness and proliferation. Furthermore, as the main proteins exposed to the host, the structure proteins induce strong antibody and cellular-mediated immune responses (Diamond *et al.*, 2008). Therefore, determination of the specific moieties or regions of the structural proteins that determine virulence and are necessary for efficient replication in the CNS could lead to development of improved subunit vaccines.

The variation in viral fitness of particles produced by astrocytes, the most abundant cell type within the CNS, may have several consequences for WNV-mediated neuropathology. For WNV-NY, the higher infectivity of particles produced by astrocytes may permit the rapid spread to more permissive cells within the CNS, such as neurons and endothelial cells, as well as infiltrating immune cells. Conversely, the less infectious nature of astrocyte-derived WNV-MAD78 may limit this strain’s ability to infect neighbouring non-astrocyte cell types. Furthermore, the production of high levels of non-infectious particles by WNV-MAD78 may provide optimal material for stimulation of the innate antiviral response within surrounding cells and the rapid activation of antigen-presenting cells. These combined effects may facilitate the rapid clearance of non-pathogenic strains, such as WNV-MAD78, from the CNS prior to widespread destruction of neurons.

Overall, our data suggest that both viral and cellular factors contribute to WNV-MAD78’s decreased infectivity and replication in astrocytes. Indeed, the differential effect of furin-like proteases on viral structural proteins may be responsible for this strain- and cell-type-specific restriction to WNV replication. Thus, the quality of viral particles produced in astrocytes, and not just the quantity, may be a determining factor in WNV replication within the CNS.

## Methods

### 

#### Cells and viruses.

Primary HBCAs (ABRI371) derived from normal human tissue were purchased from CellSystems and maintained according to the manufacturer’s instructions. Prior to use in experiments, HBCAs were grown in Dulbecco’s modified Eagle’s medium (DMEM) supplemented with 10 % FBS, antibiotic/antimycotic and non-essential amino acids (complete DMEM). All experiments with HBCAs were performed on cells passaged ≤14 times. HBMECs, obtained from K. S. Kim (Baltimore, MD, USA), were grown in RPMI 1640 supplemented with 10 % FBS, antibiotic/antimycotic, non-essential amino acids, MEM vitamins, 5 U heparin ml^−1^, NuSerum (10 %), 2 mM l-glutamine, 1 mM sodium pyruvate and 150 μg endothelial growth supplement ml^−1^ as described previously (Stins *et al.*, 2001). Vero cells were maintained in complete DMEM.

Working stocks of WNV-NY strain 3356 were generated from infectious clone pFL-WNV (Shi *et al.*, 2002). Briefly, infectious particles were recovered as previously described (Shi *et al.*, 2002), passaged once in 293 cells at a low m.o.i. and passaged subsequently in Vero cells. WNV-MAD78 was obtained from the World Reference Center for Emerging Viruses and Arboviruses (WRCEVA; Galveston, TX, USA). Lyophilized virus was resuspended in complete DMEM supplemented with 20 % FBS, amplified once in Vero cells and plaque purified. Viral stocks were amplified once in 293 cells at a low m.o.i. and working stocks were generated by passaging once in Vero cells. The titres of working stocks were determined on the respective cell lines.

#### Plasmid construction.

Constructs encoding the recombinant portions of the NY^S156P^, MAD78^P156S^, NY^MAD78(5′UTR→prM)^ and NY^MAD78(E)^ viruses were generated using the plasmids pA-NY, pA-MAD, pI-WNV-MAD(*Mlu*I-*Bam*HI), pWNV-MAD^IC^ and pFL-WNV. The plasmids pA-NY and pFL-WNV were as described previously (Shi *et al.*, 2002; Vandergaast *et al.*, 2014). The pA plasmids contained the WNV region from the 5′UTR to position 2495, which included all the structural genes. The pFL-WNV construct (Shi *et al.*, 2002) encoded the entire genome of WNV-NY strain 3356, whilst pI-WNV-MAD(*Mlu*I-*Bam*HI) encoded the T7 promoter immediately preceding nt 1–3836 of WNV-MAD78 and pWNV-MAD^IC^ encoded the T7 promoter immediately preceding the entire genome of WNV-MAD78 (Hussmann *et al.*, 2014). Plasmid pA-MAD, which encoded the structural genes of WNV-MAD, was generated by replacing the *Mlu*I/*Bgl*II fragment of MAD-AB (Suthar *et al.*, 2012) with the corresponding fragment from WNV-MAD^IC^.

To generate the pA-NY^MAD78(5′UTR→prM)^ construct, a cassette encoding WNV-MAD78 nt 500–966, WNV-NY nt 967–2495 and sequence corresponding to the vector backbone of pA-MAD (5′-CGGCAAGAACTCCGCTGTGGGAGTGGAGTCTAGAAATATTGAAAAAGCTTGGCGTAATCATGGTCATAGCTGTTTCCTGTGTGAAATTGTTATCCGC-3′) was synthesized by GenScript and inserted into pUC57. The cassette was subcloned, using Gibson Assembly Master Mix (New England Biolabs) as per the manufacturer’s directions, into the pA-MAD vector backbone (*Nsi*I and *Ngo*MIV digested).

A similar strategy was used to generate the pA-NY^MAD78(E)^ construct. A cassette consisting of WNV-NY nt 540–966, WNV-MAD78 nt 967–2468, WNV-NY nt 2470–2495 and sequence corresponding to the vector backbone of pA-NY (5′-CGGCTCTAGAGTCGAATTGAAAAAGGAAGAGTATGAGTATTCAACATTTCCGTG-3′) was synthesized by GenScript and inserted into pUC57. The cassette was subcloned, using Gibson Assembly Master Mix, into the pA-NY vector backbone (*Nsi*I and *Ngo*MIV digested).

To generate pA-NY^S156P^, pFL-WNV was used as template for PCR to amplify WNV-NY nt 1–1441 and 1421–4087. The NY 1–1441 fragment was amplified using a sense primer encoding a *Bam*HI restriction site followed by the T7 promoter immediately preceding the 5′ end of the genome and an antisense primer encoding two point mutations (A1431G and G1433A). These mutations changed residue 156 of the E protein from Ser to Pro, removing the glycosylation site. The NY 1421–4087 fragment was amplified using a sense primer containing the corresponding mutation at positions 1431 and 1433 (T1431C and C1433T), and antisense primer annealing at position 4087 (Table 1). The reaction was digested with *Dpn*I to remove any template DNA lacking the desired mutations. The resultant PCR products were assembled with the vector backbone of pFL-WNV (Shi *et al.*, 2002) (*Bam*HI and *Sph*I digested) using Gibson Assembly Master Mix.

**Table 1.** Primers used for construction of recombinant viruses

Chimera*	Amplified fragment	Primer name†	Primer sequence‡
pWNV-MAD78 ^P156S^	1102→1440	1102V	5′-AGATGATGAAGATGGAAGCAGC-3′
		1440C	5′-CAACCTGCGTGGAGTAGTTACCATGGGA-3′
	1420→3836	1420V	5′-GGTAACTACTCCACGCAGGTTGGAG-3′
		3836C	5′-GTTCTCTTGGTTGGTCCATCTCGCC-3′
pWNV-NY^S156P^	*Bam*HIT7→1441	1V§	5′-caaaggatcc*taatacgactcactatag*AGTAGTTCGCCTGTGTGAGCTGA-3′
		1441C	5′-AACCTGTGTAGGGTAGTTTCCGTGCGAC-3′
	1421→4087	1421V	5′-CGGAAACTACCCTACACAGGTTGGAGCCAC-3′
		4087C	5′-ctctagaCACTCCTCTTCTCCCT-3′
pB-NY	2495→7037	2495V	5′-gtcatcatcatcatGCCGGCAAGAGCTGAGATG-3′
		7037C	5′-GTTTCTTCTGGACTTGAGGCCTGCAACAGC-3′(Δ*Ngo*MIV)
	7024→11 029	7204V	5′-CTTGAGGCCAGCAACAGCCTGGTCACT-3′(Δ*Ngo*MIV)
		11029C	5′-gaacacaggatcttctagAGTCGAATTGAAAAAGGAAGAG-3′

* Schematics of individual chimeras are presented in Figs 5–7.

† Primers encoding the viral genome are denoted (‘V’), whilst the complementary primer for each fragment is indicated by (‘C’).

‡ Nucleotide numbering is based on the sequence from GenBank accession numbers AF404756 (NY) and DQ176636 (WNV-MAD78). Viral and non-viral sequences are in upper case and lower case, respectively. Nucleotide changes to viral sequences are underlined. Removal of restriction endonuclease sites is indicated in parentheses at the end of the primer.

§ The T7 promoter is indicated in italics.

A similar strategy was used to generate pA-MAD78^P156S^. pWNV-MAD^IC^ was used as template for PCR to amplify WNV-MAD78 nt 1102–1440 and 1420–3836. The 1102–1440 fragment was amplified using a sense primer annealing at position 1102 and an antisense primer encoding two point mutations (G1430A and A1432G). These mutations changed residue 156 of the E protein from Pro to Ser, introducing a glycosylation site. The MAD 1420–3836 fragment was amplified using a sense primer containing the corresponding mutation at position 1430 (T1430C) and 1432 (C1432T), and antisense primer annealing at position 3836 (Table 1). The reaction was digested with *Dpn*I to remove any template DNA lacking the desired mutation. The resultant PCR products were assembled with the vector backbone of pI-WNV-MAD(*Mlu*I-*Bam*HI) (*Sph*I and *Bam*HI digested) using Gibson Assembly Master Mix to generate pI-MAD78 WNV-MAD78^P156S^ intermediate.

To generate pB-MAD, WNV-MAD^(IC)^ was used as template for PCR to amplify WNV-MAD78 nt 9461–10962 using primers encoding a T9473C point mutation (Table 1), which removed the *Xba*I restriction site at this location. MAD-CG (Suthar *et al.*, 2012) was used as template for PCR to amplify WNV-MAD78 nt 2493–9488 using a primer encoding the same T9473C point mutation (Table 1). The two PCR fragments were assembled with the vector backbone of TX-CG (*Ngo*MIV and *Xba*I digested) (Suthar *et al.*, 2012) using Gibson Assembly Master Mix. The pB-NY plasmid was constructed as described previously (Vandergaast *et al.*, 2014).

All plasmids were maintained in *Escherichia coli* strain MDS42 (Scarab Genomics), a strain that has a low mutation rate, and sequenced by GeneWiz to confirm identity.

#### Generation of recombinant viruses.

The pA, pB and WNV-MAD^IC^ plasmids were digested as indicated in Table 2, and linear full-length DNA templates for *in vitro* transcription were generated by ligating the corresponding plasmid fragments as indicated in Table 2. Briefly, purified pA and pB or WNV-MAD^IC^ products were combined at a 1 : 1 molar ratio, precipitated, resuspended in water and ligated overnight. The ligated DNA was treated with 0.8 µg Proteinase K for 1 h at 37 °C, purified by phenol/chloroform extraction, precipitated and resuspended in 4–6 µl RNase-free water. The purified ligation reactions (1 µg) served as templates for *in vitro* transcription using the AmpliCap-Max T7 High Yield Message Maker kit (Cell Script). *In vitro* transcribed RNA was purified by phenol/chloroform extraction, precipitated with 5 M ammonium acetate and resuspended in water. For transfection into Vero cells, 12 µg RNA was transfected into 1×10^6^ cells using the Neon transfection system (Invitrogen) with the following settings: 1150 V, 20 ms and two pulses. Culture supernatants were collected 7 days after transfection or when cytopathic effects were visible and clarified by centrifugation at 1500 ***g*** for 5 min. Total RNA was extracted from monolayers using TRIzol Reagent (Invitrogen) as per the manufacturer’s directions. The extracted RNA was used as template for reverse transcription PCR to amplify the relevant regions of the WNV genome. The resulting PCR fragments were sequenced to confirm the presence of the inserted mutations and proper gene arrangement. The recovered viruses were amplified once in Vero cells to generate the corresponding viral stock.

**Table 2.** Restriction enzymes utilized for generating full-length DNA templates for *in vitro* transcription

Chimera	T7/WNV 5′UTR	5′ Cut	3′ Cut	WNV 3′UTR	5′ Cut	3′ Cut
WNV-NY/MAD78	pA-NY	*Mlu*I	*Ngo*MIV	pB-MAD	*Ngo*MIV	*Xba*I
WNV-MAD78/NY	pA-MAD	*Mlu*I	*Ngo*MIV	pB-NY	*Ngo*MIV	*Xba*I
WNV-NY^MAD78(5′UTR→prM)^	pA-NY^MAD78(5′UTR→prM)^	*Mlu*I	*Ngo*MIV	pB-NY	*Ngo*MIV	*Xba*I
WNV-NY^MAD78(E)^	pA-NY^MAD78(E)^	*Mlu*I	*Ngo*MIV	pB-NY	*Ngo*MIV	*Xba*I
WNV-NY^S156P^	PCR-NY^S156P^	blunt	blunt	pFL-NY	*Sph*I	*Xba*I
WNV-MAD78^P156S^	pI-MAD^P156S^	*Mlu*I	*Bam*HI	WNV-MAD^IC^	*Bam*HI	*Not*I

#### Virus titration by plaque assay.

Monolayers of Vero cells in six-well plates were washed once with Dulbecco’s PBS (DPBS; HyClone) followed by the addition of serial dilutions of viral samples. The cells were incubated in a 5 % CO_2_ incubator for 1 h at 37 °C with rocking, the inocula removed and a 0.9 % agarose/complete DMEM overlay was added. Cell monolayers were incubated for 48 h and a second overlay of agarose/complete DMEM containing 0.003 % neutral red (MP Biomedicals) was added. Plaques were counted as follows based on the appearance of plaques: WNV-NY, WNV-MAD78/NY, WNV-NY^MAD78(5′UTR→prM)^, WNV-NY^MAD78(E)^ and WNV-NY^S156P^ on days 3 and 4; WNV-NY/MAD78 on days 4 and 5; and WNV-MAD78 and WNV-MAD78^P156S^ on days 6 and 7. All titres were performed in duplicate.

#### Growth curves.

Cell monolayers were infected with WNV-NY or WNV-MAD78 at the indicated m.o.i. The amount of virus added to cultures to achieve the indicated m.o.i. was calculated using the titre of the viral stock on the respective cell type (HBCAs or HBMECs). After 1 h at 37 °C, the inoculum was replaced with complete DMEM. Culture supernatants were recovered at the indicated times and clarified by centrifugation at 1500 ***g*** for 5 min. Viral titres were determined by plaque assay on Vero cells.

#### Supernatant treatments.

Supernatants were recovered from mock- or WNV-infected (m.o.i. 0.01) HBCAs at 48 h post-infection and inactivated by exposure to UV (254 nm) for 10 min at room temperature in a Stratalinker XL-1000 (Spectronics). Complete inactivation of infectious particles was confirmed by titrating on Vero cells. UV-inactivated supernatants were diluted 1 : 1 in 2× DMEM supplemented with 20 % FBS and treated with isotype control antisera, antisera to IFN-α and IFN-β or left untreated. Fresh HBCA monolayers were exposed to UV-inactivated supernatants for 6 h at 37 °C prior to infection with WNV-NY or WNV-MAD78 (m.o.i. 0.01). After 1 h at 37 °C, the inoculum was replaced with fresh supernatant treated as described above. Culture supernatants were collected 24 h after infection, clarified by low-speed centrifugation and subjected to plaque assays on Vero cells. The amount of antisera necessary to neutralize the IFN present in supernatants recovered from WNV-infected HBCAs was determined as described previously (Hussmann *et al.*, 2013).

#### Flow cytometry.

HBCA monolayers infected with WNV-NY or WNV-MAD78 (m.o.i. 0.01) were removed from plates by trypsinization, washed twice with DPBS and fixed with 3 % paraformaldehyde. Cells were permeabilized with 0.2 % Triton X-100, blocked with 0.5 % heat-inactivated FBS and probed with WNV hyperimmune ascitic fluid (1 : 1000; WRCEVA) followed by Dylight 633 nm-conjugated goat anti-mouse IgG. For analysis, 100 000 single-cell events were collected using a FACS Canto (BD Biosciences).

#### Furin activity and inhibition.

Confluent monolayers of HBCAs or HBMECs were washed twice with DPBS and lysed in 180 µl assay buffer (100 mM HEPES, pH 7.0, 0.5 % Triton X-100 and 0.5 mM CaCl_2_). Equivalent amounts (100 µM) of the fluorogenic substrate pERTKR-amino-methylcoumarin (R&D Systems) was added to cellular lysates and fluorescence (excitation, 380 nm; emission, 460 nm) was read every 20 s for 10 min using a Spectromax M5 microplate reader (Molecular Devices). As a control, 0.2 µg recombinant furin ml^−1^ (R&D Systems) was assayed. The concentration of furin inhibitor (Dec-RVKR-CMK) needed to eliminate all detectable furin activity in HBCA lysates was determined by treating HBCAs for 48 h with serial dilutions (5–50 µM) of Dec-RVKR-CMK and assaying cell lysates as described above. For inhibition experiments, HBCAs were inoculated with WNV-NY or WNV-MAD78 (m.o.i. 0.01). After 1 h at 37 °C, the inoculum was replaced with complete DMEM in the presence or absence of Dec-RVKR-CMK at a final concentration of 50 µM. Cultures were treated with a second dose of Dec-RVKR-CMK (final concentration of 50 µM) at 24 h post-infection. Cell supernatants were collected at 48 h post-infection and clarified by centrifugation at 1500 ***g*** for 5 min. Viral titres were determined by plaque assays on Vero cells.

#### Detection and enumeration of total virus particles.

HBCAs were infected as described above. At the indicated times, culture supernatants were collected, clarified by centrifugation at 5000 ***g*** and analysed using the Virus Counter 2100 (ViroCyt) as per the manufacturer’s instructions or quantitative real-time (qRT)-PCR. For the virus counter, samples were diluted 1 : 25 in Sample dilution buffer (ViroCyt) to a total volume of 100 µl and incubated in the dark for 30 min with 50 µl Combo dye, which stains nucleic acid and protein. Two-channel fluorescence was used to detect co-localization of nucleic acid and protein. Events with simultaneous detection within both channels were defined as virus particles by ViroCyt software. For qRT-PCR, RNA was extracted from supernatants (Qiagen) of HBCAs or HBMECS infected at an m.o.i. 0.01 and 0.1, respectively, with WNV-MAD78 or WNV-NY. Total viral RNA levels were determined by qRT-PCR analysis on a Roche LC480 using Veriquest One-Step SYBR Green Master Mix (Affymetrix Biosystems) with 50 ng RNA. The total number of genomic equivalents was determined by comparison with a standard curve made from known copies of WNV. Primers utilized were: WNV-NY E (fwd) 5′-GGACCTTGTAAAGTTCCTATCTCG-3′, WNV-NY E (rev) 5′-AGGGTTGACAGTGACCAATC-3′, WNV-MAD78 E (fwd) 5′-CTGTAAGGTGCCCATTTCC-3′ and WNV-MAD78 E (rev) 5′-CCTCTTCCCACCACAATGTAG-3′. The efficiency of each primer was determined prior to standard curve generation.

#### Western blot.

Cell monolayers were washed twice with modified DPBS (HyClone) and lysed with RIPA buffer (10 mM Tris, pH 7.4, 150 mM NaCl, 0.02 % NaN_3_, 1 % sodium deoxycholate, 1 % Triton X-100, 0.1 % SDS and 1× protease inhibitors; Sigma). Lysates were subjected to SDS-PAGE and transferred to nitrocellulose membranes. Nitrocellulose membranes were blocked in 5 % non-fat dry milk diluted in Western wash buffer (PBS+0.1 % Triton X-100) and incubated with anti-WNV E (1 : 100; Fisher) or anti-actin (1 : 1000; Rockland antibodies), followed by HRP-conjugated anti-rabbit antibodies. HRP signal was visualized using electrochemiluminescence (GE Healthcare).

#### Determination of virus particle stability.

Supernatants from HBCAs and HBMECs infected with WNV-NY or WNV-MAD78 were incubated at 37 °C. Aliquots were removed at the indicated times and titrated on Vero cells.

#### Statistical analysis.

GraphPad Prism 5 was used to generate all statistical analyses. The se and significance were determined using two-tailed unpaired Student *t*-tests comparing WNV strains and chimeras.
